# Mapping neural effects of mindfulness-based cognitive therapy in ADHD using EEG microstates and machine learning models

**DOI:** 10.3389/fpsyt.2025.1670602

**Published:** 2025-11-04

**Authors:** Reza Meynaghizadeh Zargar, Sevket Hepark, Poppy L.A. Schoenberg

**Affiliations:** ^1^ Neuroscience Research Center, Tabriz University of Medical Science, Tabriz, Iran; ^2^ Radboud University Medical Center, Nijmegen, Netherlands; ^3^ Vanderbilt University Medical Center, Nashville, TN, United States

**Keywords:** ADHD, mindfulness-based cognitive therapy, EEG, microstates, machine learning, precision medicine, computational modeling methods

## Abstract

**Introduction:**

Mindfulness-based cognitive therapy (MBCT) is one of the promising treatments with no known side effects for neuropsychiatric conditions such as Attention-deficit/hyperactivity disorder (ADHD). However, the mechanism of action underlying MBCT is not clearly understood. Here, we applied resting-state EEG microstate analysis and machine learning modeling to characterize brain network dynamics in adults with ADHD exposed to MBCT.

**Methods:**

Sixty-one participants were randomized to a 12-week MBCT intervention or waitlist control (WL), with clinical assessments and EEG recordings collected pre-to-post trial. We analyzed the microstate dynamics of EEG data in different frequency bands, comparing four microstate classes (A-D), and the cross-correlation of microstate dynamics with clinical measures. Furthermore, machine learning computational techniques were applied to predict which patients can benefit more from the MBCT intervention based on their brain dynamics pre-treatment.

**Results:**

Microstate analyses revealed significant MBCT-related alterations in temporal dynamics, including increased coverage and duration of microstates A and B, as well as changes in individual explained variance in microstate A (theta band) and microstate D (alpha band). Coverage and explained variance for microstate B also showed significant changes across the full spectrum. These changes were strongly correlated with improvements in ADHD symptomatology, mindfulness skills, quality of life, and executive function across seven clinical domains. Critically, machine learning models predicted individual treatment responses with 83% accuracy using microstate dynamics.

**Discussion:**

These findings demonstrate that MBCT systematically reshapes resting-state neural microstates in ADHD, including microstate classes A, B, and D, and suggest that computational EEG biomarkers may inform precision approaches to mindfulness-based interventions.

## Introduction

Attention-deficit/hyperactivity disorder (ADHD) is one of the most prevalent neuropsychiatric conditions affecting approximately 5% of the adult population. It is defined by levels of inattention, hyperactivity, and impulsivity that are inappropriate for an individual’s developmental stage (1). Clinically, individuals with ADHD exhibit hyperactive and impulsive behaviors, and it is difficult for them to maintain sustained attention (2). Beyond these core symptoms, the disorder can also cause a range of cognitive, social, and emotional impairments (3, 4). Pharmacotherapy for ADHD is one of the most widely used treatment modalities available, which can be effective for between 70 and 85 percent of patients (5, 6). However, while these medications are generally well-tolerated, they are associated with a spectrum of side effects such as appetite suppression, weight loss, increased heart rate and blood pressure, irritability, insomnia, anger, gastrointestinal disturbances (including vomiting and stomach pain), anxiety, headache, and, in some cases, psychosis. There are even instances of sudden death reported in patients with pre-existing cardiac conditions (7). Thus, although medication remains a relatively safe option when patients are properly evaluated and monitored, there is a compelling need to explore alternative treatments, particularly for those patients who do not wish to take such medications on a long-term basis. One healthcare option may be mindfulness-based interventions that offer on-par therapeutic benefits with significantly fewer side effects, if any.

Mindfulness is understood as the deliberate focus on present-moment experiences with an attitude of acceptance and nonjudgment (8). During mindfulness practices, such as body scans, mindful movement, and seated meditation, participants develop skills to continuously return their focus to the current moment. This method is designed to enhance awareness of one’s thoughts, emotions, physical sensations, and ultimately, behavioral patterns. Emerging research on mindfulness-based interventions for adults with ADHD is promising (9, 10). For example, an initial quantitative review that included three studies on adult ADHD patients found preliminary support by demonstrating moderate to large reductions in ADHD symptoms (11). Additionally, a more recent study, which evaluated clinician-rated ADHD symptoms alongside improvements in positive mental health over six months, reported that increases in self-compassion were a key factor mediating the observed enhancements in positive mental health attributed to mindfulness-based intervention (12).

Understanding the neural underpinnings of ADHD is essential to discovering more effective therapies. Recently, atypical resting-state electroencephalogram (EEG) patterns were emphasized in a review of the  literature for those with ADHD (13). Studies focused mainly on feature extraction of EEG frequency spectra (14) or event-related potentials (ERP) (15). Although recent studies have started looking at different metrics, including the microstate properties calculated in the resting state (16, 17) and ERP (18–20) data measured in ADHD groups. Global brain activity representation of multichannel EEG recordings via microstate analysis was introduced by Lehmann et al. in 1987 (21). They consist of quasi-stable microstate patterns similar across all electrodes. This process utilizes data derived from all electrodes and time points to create a brain activity global map by calculating the amount of global field power (GFP) reflecting areas of maximal and minimal activity.

Microstates can be described in both resting-state and event-related contexts, and it has been previously reported that four canonical topographies can account for more than 70% of resting-state EEG data, usually referred to as “microstates A, B, C, and D” (22, 23). Converging evidence suggests that microstate A has been linked to auditory and language processing, microstate B to visual networks, microstate C to default mode activity, and microstate D to attentional control (24–26). These functional associations provide a useful interpretive framework, although it should be noted that EEG microstates are not direct measures of localized brain activity but rather reflect temporally coordinated dynamics of large-scale neural networks. Microstates do not ramp up smoothly, but abruptly transition after hanging out for around 80–120 ms (27). The ‘average microstate duration’, ‘coverage’, the ‘occurrence of the states’, ‘global field power/GFP’, and ‘transition probabilities between the states’ represent important metrics for microstate analysis. Despite increasing evidence on the importance of microstate functionalities in ADHD, the impact of mindfulness-based cognitive therapy/MBCT on these EEG measures is still largely unexplored. To the best of our knowledge,  this is the first study to investigate the effects of MBCT on resting-state EEG microstate dynamics data in adults with ADHD.

## Methods

### Procedure

The data collection for this study was conducted at Radboud University in the Netherlands and was approved ethically by the CMO, Arnhem-Nijmegen. The data analyzed here were part of a larger neurophysiology investigation in which EEG measures were collected concomitant to baseline (reported here), and various other cognitive behavioral paradigms (some of which have been published earlier, see (28)). Informed written consent was obtained from each patient to participate in a controlled randomized study at two time points (pre/T1 and post/T2). Participants engaged in two sessions of data collection, before and after a 12-week MBCT program for the active mindfulness treatment group, and a 12-week waiting period for the waitlist (WL) passive control group. We conducted the randomization procedure before starting the data collection in the T1 phase. In each session, participants were required to fill out clinical measures and complete an EEG recording procedure.

### Participants

**Figure 1:**
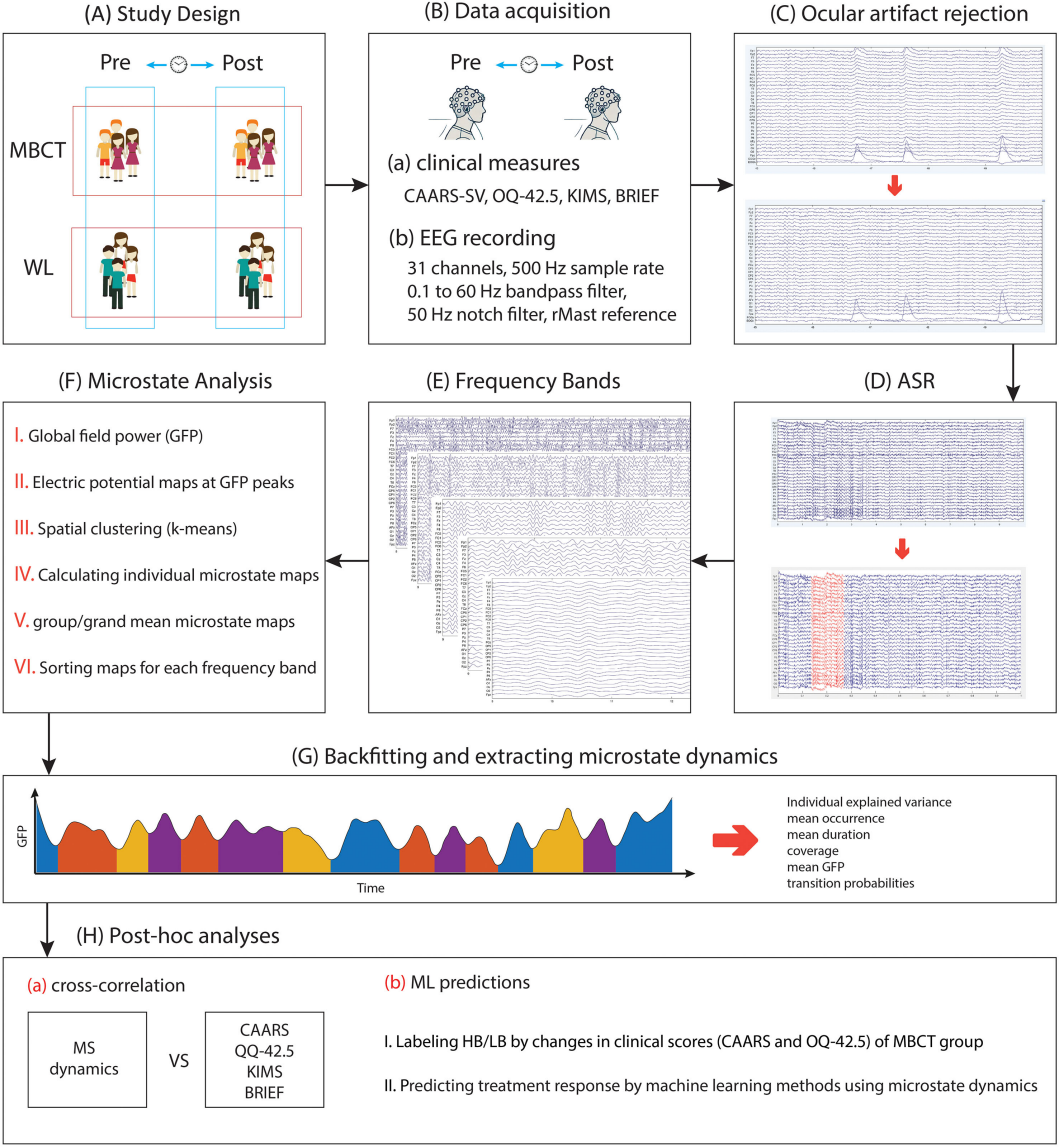
The representation of the process of the study and the conducted analysis. **(A)** Participants of the study were divided into two groups: MBCT and WL, and two time points of pre- and post-interventions. **(B)** Data acquisition of clinical and EEG data. **(C)** removing ocular artifacts using a regression-based method. The image below shows changes after the ocular artifacts rejection procedure. **(D)** removing bad segments using the artifact subspace reconstruction (ASR) method. The red segment in the image below represents the portion that was removed during this procedure. **(E)** segmenting EEG recordings into different frequency bands. **(F)** conducting microstate analysis. **(G)** backfitting and extracting microstate dynamics. **(H)** conducting *post-hoc* analysis, including cross-correlation and machine learning (ML) techniques.

Initially,  61 adult ADHD patients were included from the outpatient unit at Radboud University Nijmegen Medical Centre. The patients were randomly divided into two groups: 32 of them received MBCT, and 29 individuals were placed on a waitlist (**Figure 1A**). Eleven patients (6 in the MBCT group and 5 in the WL group) did not attend the post-treatment (T2) testing session. One individual did not complete the full 12-week MBCT program, and another one engaged in mindfulness training outside of the study protocol, whereby both were excluded from the study. Nine other patients dropped out of the study due to scheduling and organizational constraints. This attrition led to a final sample size of 50 patients, with n=26 patients in the MBCT group and n=24 patients in the WL group. Inclusion criteria were a primary diagnosis of ADHD (as confirmed by three psychiatrists following DSM-IV-TR criteria) and participants’ ages between 18 and 65 years. Furthermore, key exclusion criteria included a history of substance abuse or dependence within the past six months, co-morbid conditions, such as the presence of psychotic, borderline, antisocial, and/or other behavioral disorders, and learning difficulties. Of these 50 patients, 31 (15 in the MBCT group, and 16 in the WL group) were currently on pharmacotherapy. Of these, 19 were treated with methylphenidate-based drugs, 8 with dextroamphetamine-based drugs, and 4 with antidepressants. The other 19 patients were given no medication. For participants on stimulant medications, dosages were stabilized two weeks before the start of the study, while participants on non-stimulants were stabilized four weeks prior; no changes were made to medications during the study.

### Clinical measures

Clinical measures were conducted pre-and-post randomized trial: (i) Conners’ Adult ADHD Self-rating Scale (CAARS-SV) which measures global DSM-IV ADHD symptoms, ‘inattention’, and ‘hyperactivity– impulsivity’ subdomains (29); (ii) Quality of Life (OQ-42.5) test which, besides the global score, measures ‘interpersonal relations’, ‘symptom distress’, and ‘social role’ subdomains (30); (iii) Kentucky Inventory of Mindfulness Skills (KIMS) which includes four mindfulness skills: ‘observe’, ‘describe’, ‘act with awareness (AWA)’, and ‘accept without judgment (AWoJ)’ (31). It is worth noting that none of the patients had any exposure or experience of mindfulness/yoga/etc before the study. (iv) Behavior Rating Inventory of Executive Function (BRIEF), which is a standardized assessment tool used to evaluate executive function behaviors (32). It includes 86 items and is categorized into two main indices. The Behavioral Regulation Index (BRI) has three subdomains, including inhibition (INHI), shifting (SHIFT), and emotional regulation (EMOT). The Metacognition Index (MI) includes initiation (INAT), working memory (WM), planning/organization (PLAN), organization of materials (ORGA), self-monitor (SELF), and Task Monitor (TASK). Furthermore, the scores of MI and BRI were summed to calculate the Global Executive Composite (GEC). Severe impairments of executive functions are identified by higher scores on the BRIEF.

### MBCT intervention

The MBCT program is a modified version of an established protocol that was initially designed for depressive disorders. In this program, participants engage in structured exercises in 12 weekly sessions, which take three hours in each session (33, 34). We used Workbooks, including psycho-educative modules designed specifically for ADHD patients. The program required an average time of 30–45 minutes of self-practice per day, which was guided by compact disks (CDs). A trainer was responsible for monitoring the patient’s maintenance of self-practice. The course was directed by a specialist psychiatrist in ADHD, with 9 years of experience in MBCT training at the time of the study.

### Electrophysiological recording

During EEG recording periods, participants were instructed to keep their shoulders and forehead relaxed and avoid doing eye movements or blinking as much as possible. EEG signals were collected using Brain Vision Recorder 1.03 software and QuikAmps 72 equipment (http://BrainProducts.com), captured from 30 Ag/AgCl active electrode sensors featuring built-in noise cancellation technology (actiCAP: Brain Products) positioned following the 10–10 electrode placement system (locations: Fp1, Fp2, AFz, F7, F3, Fz, F4, F8, FC5, FC1, FCz, FC2, FC6, T7, C3, Cz, C4, T8, CP5, CP1, CP2, CP6, P7, P3, Pz, P4, P8, O1, Oz, O2). An average online reference was applied and later adjusted to the right mastoid during offline processing. The ground electrode was placed on the forehead. Vertical and horizontal eye movements were measured using bipolar electrooculogram recordings obtained from Ag/AgCl cup electrodes positioned above and below the left eye and 1 cm from the outer corners of each eye, respectively. Impedance levels were kept below 10 KΩ. The electrical activity was continuously recorded at a sampling frequency of 500 Hz, with a band-pass filter set to 0.1–100 Hz (**Figure 1B**).

### Signal analysis of microstate classification

The analysis of the signal in our study was initiated by a 0.1 Hz high-pass filter, a 60 Hz low-pass filter, and a 50 Hz notch filter. To remove ocular artifacts, we used a regression-based procedure that leverages EOG channels, as described by Gratton et al. (35) and Croft & Barry (36) (a Python tutorial is available at https://mne.tools/stable/auto_tutorials/preprocessing/35_artifact_correction_regression.html#footcite-grattonetal1983) (**Figure 1C**). After eliminating these artifacts, the artifact subspace reconstruction (ASR) method (37) was employed via an EEGLAB plugin to identify and remove additional artifacts and problematic data segments, using default parameters (including a maximum 0.5-second window standard deviation of 20) (**Figure 1D**).

Resting-state EEG microstate analysis was subsequently performed in MATLAB 2024b with EEGLAB plugin version 2024.2 and the MICROSTATELAB plugin, following the guidelines outlined by Kalburgi et al. (38). We analyzed EEG microstates separately in delta (0.5 Hz – 4 Hz), theta (4 Hz – 8 Hz), alpha (8 Hz – 12 Hz), beta (13 Hz – 30 Hz), gamma (30 Hz – 60 Hz) frequency bands, and also in full spectrum (0.5 Hz – 60 Hz) (**Figure 1E**).

Microstate dynamics were derived by initially calculating the Global Field Power (GFP) of the resting-state data for each participant (**Figure 1F**). Topographic maps were then constructed based on the Global Field Power/GFP peaks because scalp topographies are rather stable around these peaks and show the highest signal-to-noise ratio. Those maps were clusterized for each subject with the k-means algorithm. Global Field Power/GFP is the measure of the spread of potential across all electrodes at a given instant for a given time, which is defined as follows:


$$GFP\left(t\right)=\sqrt{\frac{\sum_{i=1}^{n}{\left(V_{i}\left(t\right)-V_{mean}\left(t\right)\right)}^{2}}{n}}$$

Where *i* denotes the electrode, *n* represents the total number of electrodes, *V* corresponds to the measured voltage, and *t* refers to the specific time point.

Grand mean maps were constructed by averaging maps within each group over participants. We chose the four-class microstate solution for this work as this solution approximated the most commonly reported microstate maps found in the literature, making it easier to compare our data to previous research. Maps were then automatically indexed and graded as microstates A, B, C, and D according to a template from Koenig et al. (39). The grand mean maps were next back-fitted on the single datasets to estimate descriptive parameters for each microstate classification: Individual explained variance, mean Global Field Power/GFP, occurrence, duration, coverage, and transition probabilities (**Figure 1G**). Occurrence is the average number of appearances of a microstate per second; Duration is the mean time (in milliseconds) before x microstate switches to another status; Coverage is the percentage of the total record time occupied by a microstate.

We calculated the complexity of the sequence of microstate classes in EEG data by the method proposed by Tait et al. (40). This method implements the Lempel–Ziv complexity (LZC), which is defined as the number of various subsets in a set of values (microstate classes). A sequence with a low number of repeated subsets is considered to have low complexity. To calculate the Lempel–Ziv complexity/LZC, we first extracted the microstate data backfitted in each time point for each subject, then we used a Python code to measure the number of distinct subsets of microstate classes that repeat in the first 250 microstates.

For analysis of between-groups (MBCT vs. WL) and changes between pre- and post-assessments, we used a multi-factor between- and within-subjects repeated measures ANOVA. The between-subject factor was Group (MBCT compared to WL), and the within-subject factor was Time (pre compared to post). In all  statistical tests, p< 0.05 was considered to be significant.

### *Post-hoc* analyses: cross-correlation and machine learning predictions

We were also interested in the relationship between changes in microstate dynamics and changes in clinical measures after 12 weeks of mindfulness practice. We examined the normal distribution of each pair in correlation comparison using the Shapiro-Wilk test. For normally distributed features, we used Pearson correlation; in another case, we implemented Spearman correlation comparison (**Figure 1H**). The number of subjects in correlation analysis varied between 13 and 19 in the MBCT group and 20 to 22 in the WL group (due to missing values).

We implemented machine learning techniques to predict which patients would potentially garner more benefit from the mindfulness program based on their baseline data (**Figure 1H**). To do so, we first labeled patients as “high-benefitters”/HB and “low-benefitters”/LB using their clinical measures before and after MBCT. CAARS global and Quality of Life (OQ-42.5) global scores were used. We calculated increment change [post-pre] values for each patient and aggregated CAARS values after negation with OQ-42.5 values. Higher values of CAARS indicate worse ADHD symptoms, and higher values of QQ-42.5 indicate better quality of life. So, we negated all values of CAARS before aggregating with QQ-42.5 values in order to reach a unified approach. Detailed data for categorization are prepared in **Table 1**. Patients with scores lower than the median were categorized as high-benefitters, and patients with scores higher than the median were categorized as low-benefitters. Microstate dynamics were used as machine learning features to train the model. We implemented 6-fold cross-validation and three machine learning methods, including binary logistic regression (LR), support vector machine (SVM), and random forest (RF), for the treatment response prediction. The clinical measures of some individuals were missing (because they did not complete the measures or for other reasons), which resulted in 18 subjects in the machine learning prediction model. We reported metrics of the confusion matrix for machine learning models, including accuracy, sensitivity, and specificity. These metrics were calculated using true positive (TP, truly labeled as HB), true negative (TN, truly labeled as LB), false positive (FP, falsely labeled as HB), and false negative (FN, falsely labeled as LB) values. Accuracy ((TP+TN)/(TP + TN + FP + FN)) tells us what percent of the data is labeled correctly as LB and HB. Sensitivity (TP/(TP + FN)) tells us what percent of HB subjects are correctly labeled as HB. Specificity (TN/(TN + FP)) tells us what percent of LB subjects are correctly labeled as LB.

**Table 1:** The values of CAARS and QQ-42.5 were used for categorizing patients in the MBCT group as high-benefitters and low-benefitters.

Group	QQ-42.5	CAARS	QQ-42.5 - CAARS
High-benefitters	10	-4	14
	5	-5	10
	4	-5	9
	-5	-13	8
	3	-5	8
	0	-1	1
	3	2	1
	-1	0	-1
	-4	-2	-2
Low-benefitters	0	3	-3
	-12	-9	-3
	-16	-12	-4
	-1	4	-5
	-17	-10	-7
	-13	-5	-8
	-18	-6	-12
	-13	1	-14
	-22	-2	-20

## Results

### Clinical measure

Groups of the study were matched in terms of age, sex, and medication status. The results of repeated-measure ANOVA showed a significant change in clinical measures of CAARS-SV (ADHD symptoms), OQ-42.5 (quality of life), and KIMS (mindfulness skills) between groups of the study. Discussion of clinical measures from the broader study cohort, overlapping with the sample examined here, has been reported previously (41).

**Figure 2:**
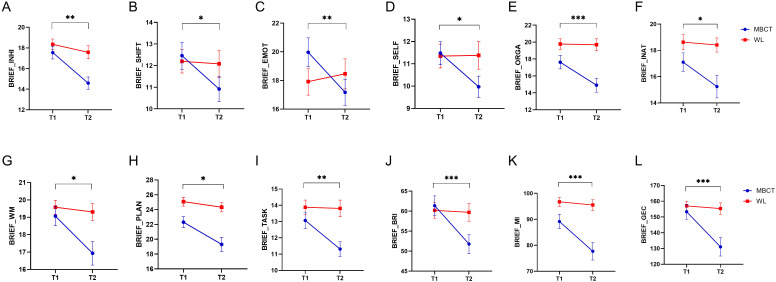
The results of MBCT on the Behavior Rating Inventory of Executive Function (BRIEF) measure. The y axes in each figure represent the raw data of each measure. **(A)** inhibition (INHI). **(B)** shifting (SHIFT). **(C)** emotional regulation (EMOT). **(D)** self monitor (SELF). **(E)** organization of materials (ORGA). **(F)** initiation (INAT). **(G)** working memory (WM). **(H)** planning/organization (PLAN). **(I)** Task Monitor (TASK). **(J)** Behavioral Regulation Index (BRI). **(K)** Metacognition Index (MI). **(L)** Global Executive Composite (GEC). Blue lines are related to the MBCT group, and red lines are related to the WL group. Bar lines represent the standard error of the mean (SEM). *P < 0.05, **p < 0.01, ***p < 0.001.

The analysis of BRIEF (executive functioning) clinical scores, as depicted in **Figure 2**, indicated that there were a significant decrease in all three cumulative scores of Behavioral Regulation Index (F_1, 47_ = 14.54, p< 0.001, η² =0.23), MI (F_1, 53_ = 14.93, p< 0.001, η² =0.22), and GEC (F_1, 47_ = 18.72, p< 0.001, η² =0.28), and 9 subscores of INHI (F_1, 53_ = 9.09, p = 0.004, η² =0.14), SHIFT (F_1, 47_ = 6.58, p = 0.014, η² =0.12), EMOT (F_1, 53_ = 11.66, p = 0.001, η² =0.18), SELF (F_1, 53_ = 5.26, p = 0.026, η² =0.09), INAT (F_1, 54_ = 5.02, p = 0.029, η² =0.08), WM (F_1, 53_ = 6.21, p = 0.016, η² =0.1), PLAN (F_1, 54_ = 6.04, p = 0.017, η² =0.1), TASK (F_1, 53_ = 7.92, p = 0.007, η² =0.13), ORGA (F_1, 53_ = 16.99, p< 0.001, η² =0.24), MI (F_1, 53_ = 14.93, p< 0.001, η² =0.22), GEC (F_1, 47_ = 18.72, p< 0.001, η² =0.28).

### Microstate dynamics

**Figure 3:**
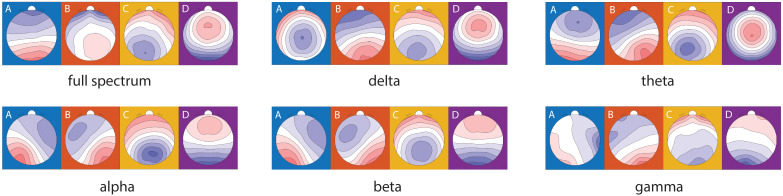
Microstate topographical maps of the grand mean of all subjects in two groups and two time points in different frequency bands.

**Figure 4:**
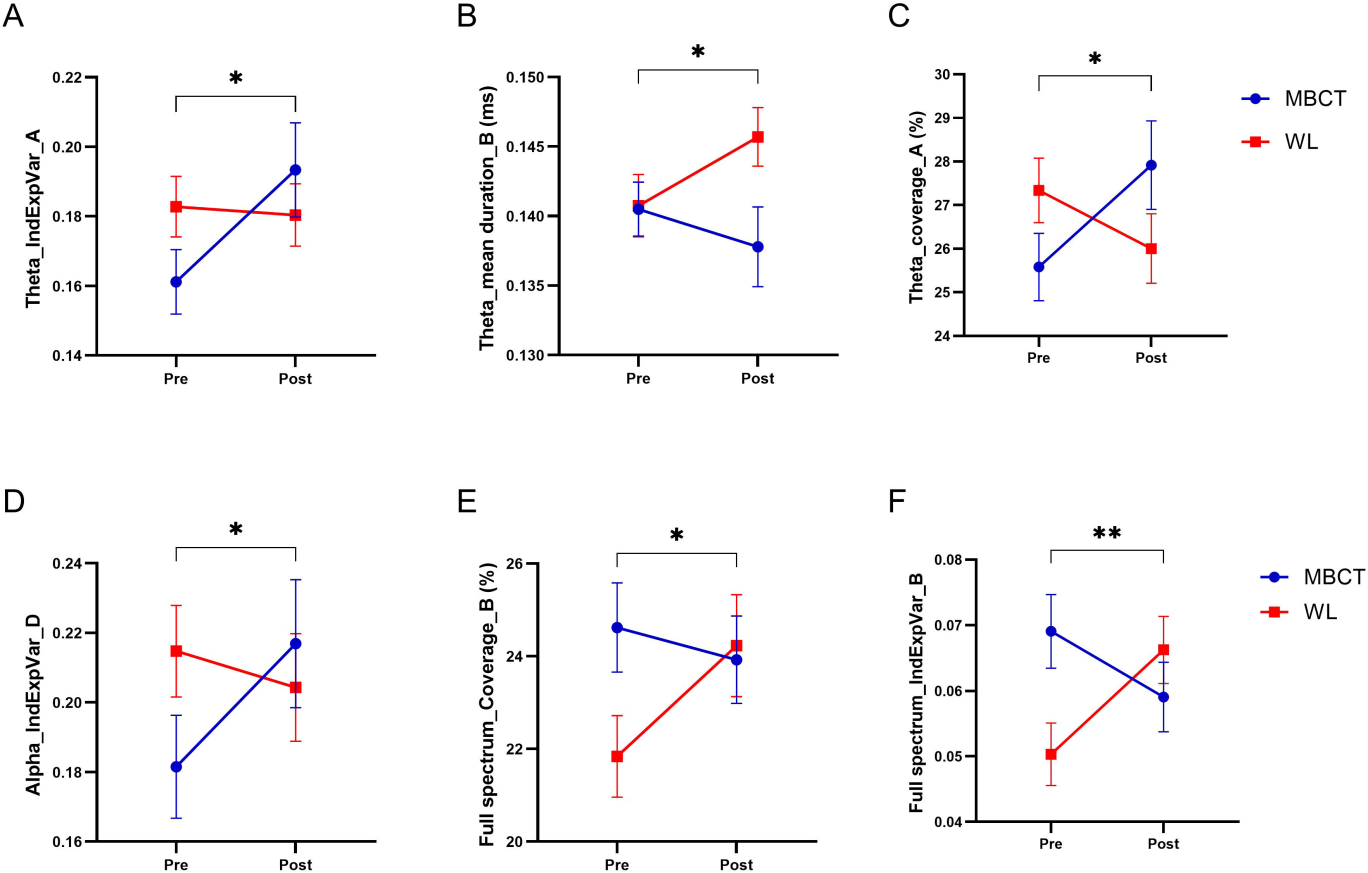
The results of MBCT on microstate temporal dynamics of ADHD patients. **(A)** The individual explained the variance of microstate A in the theta frequency band. **(B)** Mean duration of microstate B in the theta frequency band. **(C)** Coverage of microstate A in theta frequency band. **(D)** The individual explained variance of microstate D in the alpha frequency band. **(E)** Coverage of microstate B in the full spectrum. **(F)** The individual explained variance of microstate B in the full spectrum. Each bar represents the mean value of the data, and bar lines represent the standard error of the mean (SEM). *P < 0.05, **p < 0.01.

The topographic microstate maps of grand means are shown in **Figure 3** for each frequency band. The results of statistical analysis showed that there was a significantly group×time effect between MBCT and WL groups which shows an increase in coverage of microstate A (F_1, 45_ = 6.12, p = 0.017, η² =0.12), a decrease in mean duration of microstate B (F_1, 43_ = 7.06, p = 0.011, η² =0.14), and an increase in individual explained variance of microstate A (F_1, 46_ = 6.73, p = 0.013, η² =0.13) in the theta frequency band (**Figures 4A-C**) in the MBCT group compared to WL. We also observed a significant group×time effect between groups of the study which shows a decrease in the coverage of microstate B (F_1, 43_ = 5.95, p = 0.019, η² =0.12) and a decrease in individual explained variance of microstate B (F_1, 48_ = 11.7, p = 0.001, η² = 0.19) when considering the whole spectrum of frequency bands in the MBCT group compared to WL (**Figures 4E, F**). Our results indicated a significant increase (F_1, 43_ = 6.53, p= 0.014, η² = 0.13) of the individual explained variance of microstate D in the MBCT group compared to WL in the alpha frequency band (**Figure 4D**).

**Figure 5:**
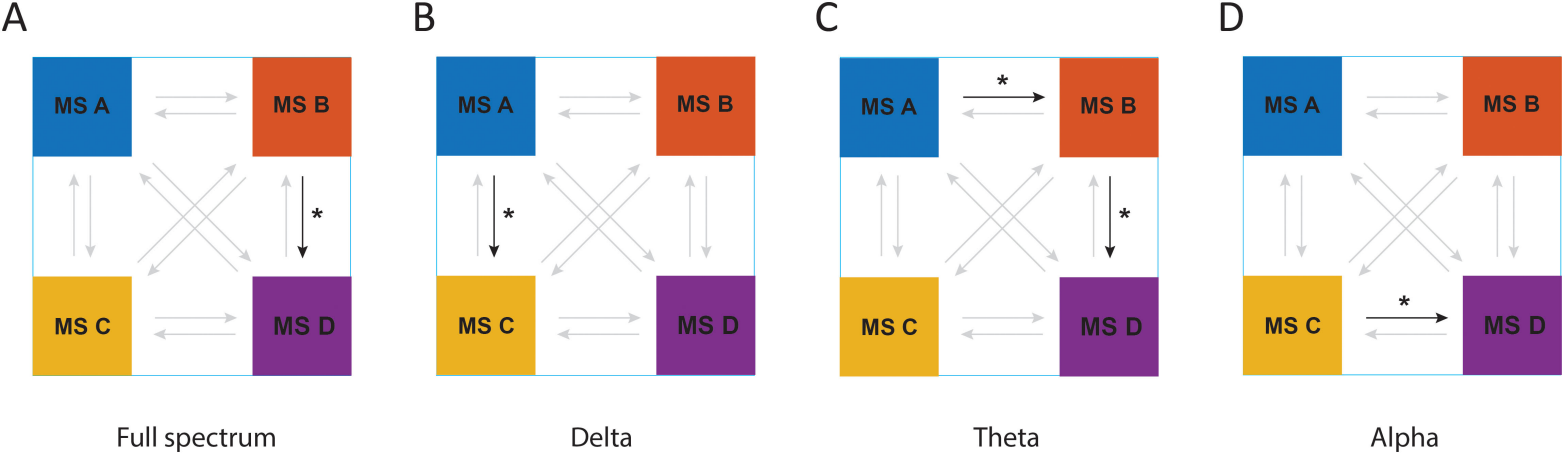
The results of MBCT on transition probabilities of microstates in ADHD patients in **(A)** full spectrum, **(B)** delta frequency band, **(C)** theta frequency band, and **(D)** alpha frequency band. Arrows with bold colors represent a meaningful transition probability. *P < 0.05.

As represented in **Figure 5**, the analysis of transition probabilities between different microstate classes revealed that there was a significant difference between groups of the study in transition probabilities of microstate B to microstate D in full spectrum (F_1, 43_ = 5.71, p = 0.021, η² =0.11); microstate A to microstate C in the delta frequency band (F_1, 44_ = 6.41, p = 0.015, η² =0.12); microstate A to microstate B (F_1, 44_ = 6.43, p = 0.015, η² =0.12) and microstate B to microstate D (F_1, 45_ = 4.17, p = 0.047, η² =0.08) in theta frequency band; and microstate C to microstate D in alpha frequency band (F_1, 45_ = 5.32, p = 0.026, η² =0.1).

The results of repeated measures ANOVA indicated that there was no significant within-subjects difference in Lempel–Ziv complexity/LZC values in any of the frequency bands.

### *Post-hoc* analyses: cross-correlation

**Figure 6:**
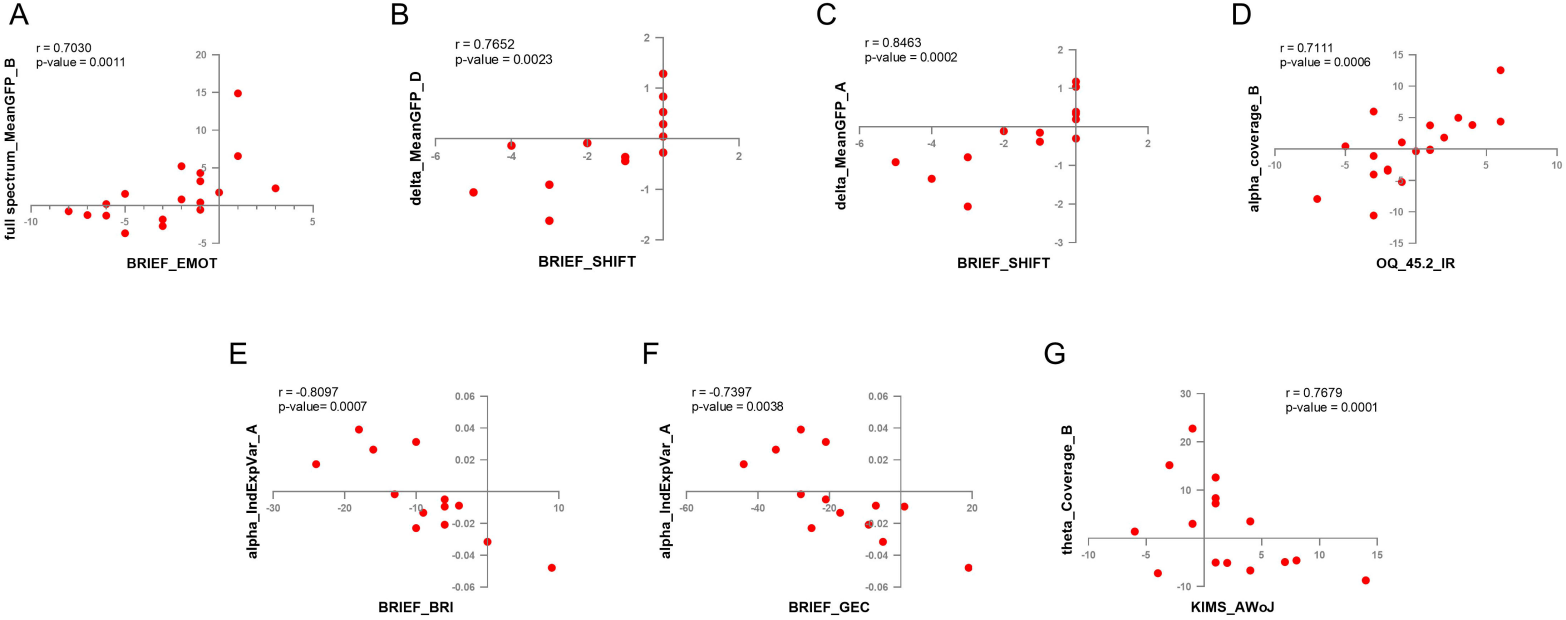
The results of cross-correlation between EEG microstate dynamics and clinical measures. **(A)** Coverage of microstate B in the alpha frequency band and OQ-42.5 interpersonal relations (IR) scores. **(B)** The individual explained variance of microstate A in the alpha frequency band and the BRIEF Behavioral Regulation Index (BRI) scores. **(C)** The individual explained variance of microstate A in the alpha frequency band and BRIEF Global Executive Composite (GEC) scores. **(D)** Mean GFP of microstate A in the delta frequency band and BRIEF shifting (SHIFT) scores. **(E)** Mean GFP of MS D in the delta frequency band and BRIEF SHIFT scores. **(F)** Mean GFP of microstate B in full spectrum and BRIEF emotional regulation (EMOT) scores. **(G)** Coverage of microstate B in the theta frequency band and KIMS accept-without-judgement (AWoJ) scores.

The results of the correlation between clinical score increment change and microstate dynamics increment change (calculated as post-pre values) in the different frequency bands indicated that there was no correlation when considering the MBCT and WL groups together. However, when splitting the dataset by treatment Group, we found no significant correlations between these values within the WL group, but there were various strong correlations (r ≥ 0.7) between clinical scores and microstate dynamics in the MBCT group only. Specifically, in seven pairs of measures (1): Mean global field power/GFP of microstate A in the delta frequency band and BRIEF shifting scores (r = 0.8463, p = 0.0002) (2); Mean global field power/GFP of microstate D in the delta frequency band and BRIEF shifting scores (r = 0.7652, p = 0.0023) (3); Coverage of microstate B in the theta frequency band and KIMS Act Without Judgement scores (r = 0.7679, p = 0.0002) (4); Individual Explained Variance of microstate A in alpha frequency band and BRIEF Global Executive Composite scores (r = -0.7397, p = 0.0038) (5); Individual Explained Variance of microstate A in alpha frequency band and BRIEF Behavioral Regulation Index scores (r = -0.8097, p = 0.0008) (6); Coverage of microstate B in alpha frequency band and OQ_45.2 Interpersonal Relations score (r = 0.7111, p = 0.0006) (7); Mean global field power/GFP of microstate B in full spectrum (0.1 to 60 Hz) and BRIEF emotional regulation scores (r = 0.7030, p = 0.0011). The scatter plots of the correlation comparisons can be found in **Figure 6**. Four of these pairs (**Figures 6D-G**) had a normal distribution and were analyzed by Pearson correlation, and three other pairs (**Figures 6A-C**) were calculated by Spearman correlation. The p-value for all these calculations was lower than 0.01. It is worth noticing that the Coverage of microstate B and individual explained variance of microstate A each appeared in two correlations (**Figures 6D-G**), and the most observed clinical measure that correlated with microstate dynamics was the BRIEF indexing executive functioning.

### Machine learning predictions

**Figure 7:**
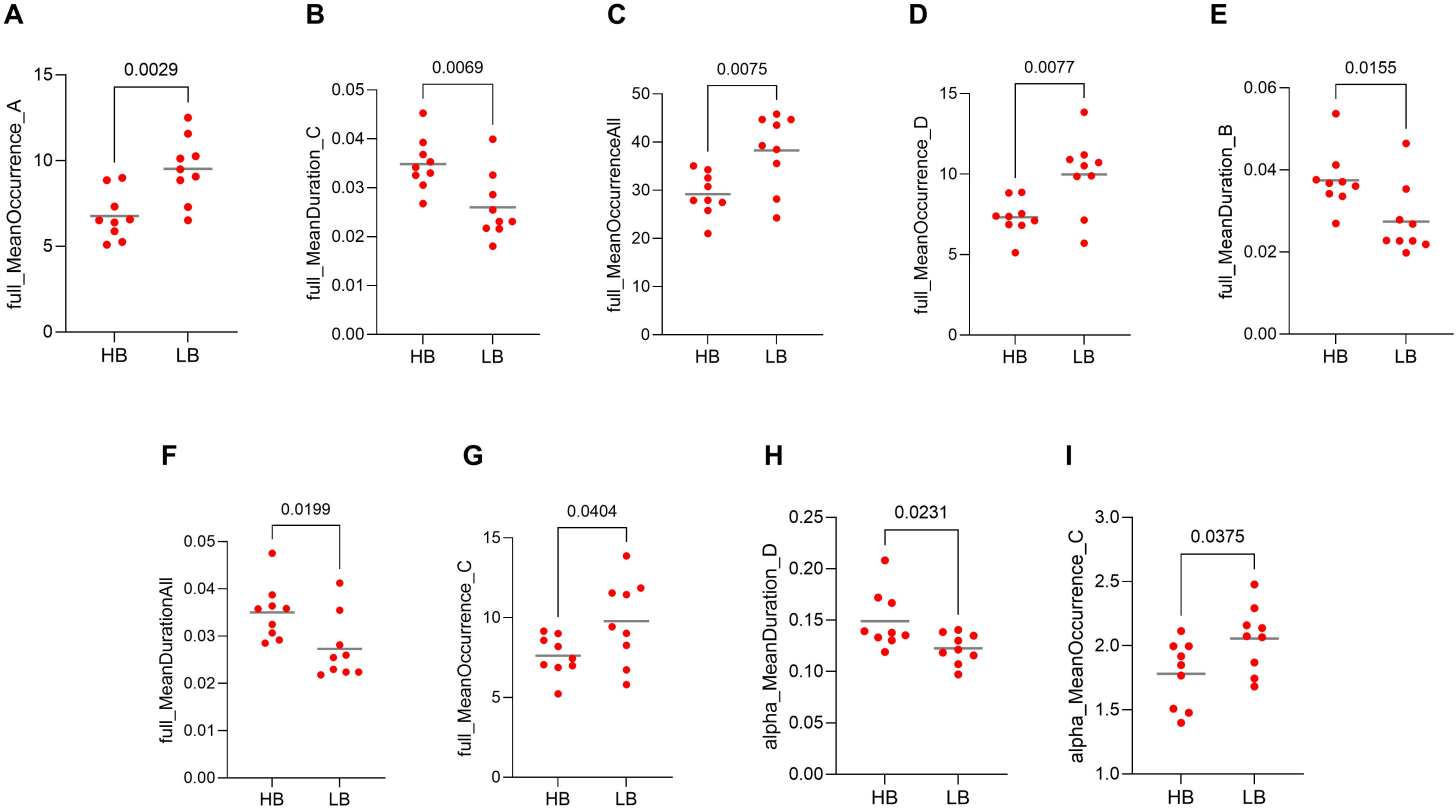
Representation of features used in machine learning (ML) model training, including microstate dynamics. Values compared between high-benefitters (HB) and low-benefitters (LB) in pre-intervention data of the MBCT group. **(A)** occurrence of microstate A in full spectrum, **(B)** duration of microstate C in full spectrum, **(C)** occurrence of all microstates in full spectrum, **(D)** occurrence of microstate D in full spectrum, **(E)** duration of microstate B in full spectrum, **(F)** duration of all microstates in full spectrum, **(G)** occurrence of microstate C in full spectrum, **(H)** duration of microstate D in alpha frequency band, **(I)** occurrence of microstate C in alpha frequency band.

The results of the t-test between those patients classed as high-benefitters and low-benefitters indicated that there were significant differences in nine microstate dynamics between these classifications. Seven of these microstate dynamics were in full spectrum band including the occurrence of microstate A (p = 0.0029, **Figure 7A**), duration of microstate C (p = 0.0069, **Figure 7B**), the occurrence of all microstates (p = 0.0075, **Figure 7C**), the occurrence of microstate D (p = 0.0077, **Figure 7D**), the duration of microstate B (p = 0.0155, **Figure 7E**), duration of all microstates (p = 0.199, **Figure 7F**), the occurrence of microstate C (p = 0.0404, **Figure 7G**) and two of them were in alpha frequency band including the duration of microstate D (p = 0.0231, **Figure 7H**) and the occurrence of microstate C (p = 0.0375, **Figure 7I**). These are our machine learning microstate features.

**Figure 8:**
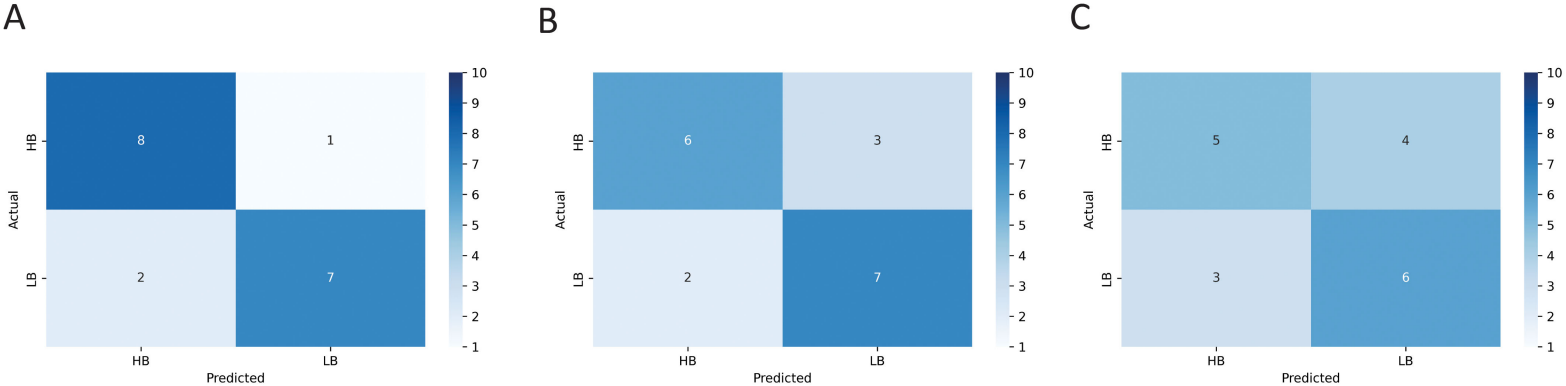
The confusion matrix resulted from training the **(A)** logistic regression (LR), **(B)** support vector machine (SVM), and **(C)** random forest (RF) models using microstate features represented in **Figure 7**.

The results of the LR, SVM, and RF machine learning models indicated an accuracy of 0.83, 0.72, and 0.61, respectively, when implementing machine learning microstate features. In order to visualize and evaluate the performance of the classification model we used, the confusion matrix of the machine learning models implemented in this study is shown in **Figure 8**. We also summarized the output metrics of machine learning methods, including accuracy, sensitivity, and specificity, in **Table 2**.

**Table 2:** The results of machine learning methods used for predicting the treatment response of patients in the MBCT program.

Classification	Accuracy	Sensitivity	Specificity
LR	0.83	0.88	0.77
SVM	0.72	0.66	0.77
RF	0.61	0.55	0.66

LR, binary logistic regression; SVM, support vector machine; RF , random forest.

## Discussion

We investigated the neural and clinical effects of mindfulness-based cognitive therapy (MBCT) in adults with ADHD, integrating resting-state EEG microstate analysis and machine learning prediction models. Participants were randomly assigned to the MBCT or waitlist (WL) control groups, with EEG and clinical assessments collected pre- and post-intervention. We conducted additional *post hoc* analyses (1): exploring correlations between microstate dynamics and clinical outcomes, and (2) applying machine learning techniques to predict individual treatment responsiveness based on baseline data. The results of our study show that microstate patterns of brain activity, reflecting moment-to-moment brain dynamics, can be modified by MBCT, a non-pharmacological treatment with no known side effects, in adults with ADHD. Interestingly, these pattern modifications are linked with clinical measures and highlight their role in cognitive performance, such as better executive functioning and greater emotional control. These findings suggest that MBCT improves ADHD symptoms, perhaps by reshaping large-scale brain network dynamics, as captured by EEG microstates, providing support for the use of neuromodulatory interventions such as mindfulness and neurofeedback. We also used microstate patterns to predict if patients can benefit from MBCT before going through the treatment, reducing financial burden and unnecessary engagement in an extra treatment process.

To our knowledge, this is the first study to assess changes in EEG microstates following MBCT in ADHD patients, and the first to incorporate machine learning models for predicting therapeutic benefit from MBCT using microstate and clinical features. The 12-week interval between baseline and post-treatment recordings allowed us to assess longitudinal changes. Crucially, the inclusion of a WL control group enabled us to distinguish treatment effects from temporal changes, with repeated measures ANOVA confirming significant group-by-time interactions in microstate dynamics. Importantly, prior research supports the test–retest reliability of core microstate parameters, encompassing duration, occurrence, and coverage metrics (42); enhancing confidence in the observed intervention-related effects. However, lower reliability has been reported for microstate transition metrics, which were not central to our findings.

We used a regression-based ocular artifacts removal method and implemented another Artifact Subspace Reconstruction/ASR artifact reduction method before analyzing EEG microstate dynamics. Other studies that have measured EEG microstates of ADHD patients have implemented other methods of preprocessing, such as independent component analysis/ICA (16, 17, 43) or manual artifact rejection (43, 44), which can affect the results and interpretations of findings. Studies have shown that there is some differentiation with regard to analytical outcomes using different preprocessing approaches, especially in low-frequency spectral features (45). However, a recent study indicated that microstate dynamics are robust to artifacts, regardless of how the data is preprocessed (46). This indicates that we can compare the results of our study with other published studies, even where they have used different preprocessing approaches.

The results of our analysis showed a modification in the clinical measures of ADHD post-MBCT. As depicted in **Figure 2**, BRIEF scores decreased, indicating improvements in the domains of inhibition, shifting, emotional regulation, initiation, working memory, planning/organization, organization of materials, self-monitor, and task monitor following exposure to the MBCT. Previous studies report similar findings with regards to improved executive functioning after mindfulness intervention. For example, Virone reported symptom amelioration after a mindfulness intervention for ADHD patients using the BRIEF test (47). In another study, MBCT was associated with significantly lower ADHD symptoms compared to treatment as usual. In the same study, interestingly statistically significant improvements were not observed in executive functions immediately after MBCT exposure, but were observed after a 6-month follow-up (48).

There has been interest in recent years about examining microstate dynamics of resting state EEG in patients with psychiatric disorders, of which there are a few studies in ADHD patients. Previous work suggests that resting-state EEG microstates are significantly affected by ADHD. A recent review article summarized studies that evaluated resting-state and event-related microstates of ADHD patients (49). They have reported 13 studies, including three in resting-state, in their review.

By conducting a microstate analysis on the resting-state EEG recordings of patients with ADHD, Férat et al. discovered five microstate classes. They conducted their analysis on two datasets, both between ADHD and healthy control subjects. According to their results, ADHD participants showed longer durations of microstate D and decreased duration and coverage of microstate A, which had an inverse correlation with inattention scores. Importantly, the results for microstate D were robust as they were replicated in another dataset (17). In another study by Luo et al., four microstate maps were compared between groups of children with ADHD and healthy controls. The authors reported that the coverage of microstate C was lower in the ADHD group, and the duration and contribution of microstate D were also higher in the ADHD group compared to the healthy control group (44). Adding to these outcomes, Wu et al. compared healthy participants with ADHD persistent (ADHD-P) and ADHD remission (ADHD-R). They identified four microstates across the dataset and found that both ADHD groups had higher durations of microstate C compared with the healthy control group. Furthermore, the ADHD-R group had higher coverage of microstate C, increased transition probabilities from microstate C to D, and decreased transition probabilities from microstate D to C (16). Comparing the prior finding with the current study shows the possible role of microstate A in ADHD symptoms. Our findings show an increase in the coverage of microstate A after MBCT, which was shown to be reduced in ADHD patients by Férat et al. (17).

Moreover, research carried out by Leon et al., examining EEG microstates in ADHD patients, has recognized four primary maps common to all participants in their investigation (43). In children with ADHD, microstate B exhibited a significant reduction in coverage relative to the healthy control group. Additionally, the analysis of transition probability between groups revealed a higher transition from state C to state D. In another study, Piao et al. used resting-state microstate analysis to assess neurobiological markers in three groups of study, including healthy controls, ADHD patients with sleep problems (ADHD-SP), and ADHD patients without sleep problems (ADHD-NSP) (50). Their results indicated that both clinical ADHD groups had significantly lower occurrence of microstate D and reduced transition probability from microstate C to D compared with healthy controls. Furthermore, the ADHD without sleep problems group (ADHD-NSP) showed a lower duration of microstate A and reduced transition probabilities from microstate D to C.

Looking at meditation studies more broadly, such as the effects of different kinds of meditation, including mindfulness practice, shows compelling modulation of EEG microstate dynamics. For example, Zarka et al. evaluated changes in microstate dynamics in resting-state EEG in individuals after mindfulness-based stress reduction (MBSR) training versus those in a waitlist control group. They reported that the MBSR group displayed lower duration, coverage, and occurrence of microstate C compared with controls (51). Below we synthesize the aforementioned evidence base with how it connects to our study findings (**Table 3**).

**Table 3:** Summary of the results of EEG microstate dynamics in other germane studies compared to the current study.

Study	Details	Groups	MS classes	In. exp. Var.	Occurrence	Duration	Coverage	Mean GFP	Transition probabilities	Reference
Férat et al. (2022)	Dataset 1: eyes open, 1–100 Hz, 31F/35M, mean age = 34 Dataset 2: eyes open, 1–100 Hz, 12F/10M, mean age = 32	ADHD; HC	5			D↑ A↓	A↓			(17)
Luo et al. (2023)	eyes closed; 1–45 Hz; 38F/123M; 8–15 years	ADHD; HC	4			D↑	C↓		(A&C)↓ (B&D)↑	(44)
Wu et al. (2024)	eyes closed; 0.5–45 Hz; 7F/21M; 18–27 years	ADHD-P; ADHD-R; HC	4			C↑	C↑*		(C→D)↑* (D→C)↓*	(16)
Leon et al. (2024)	eyes open; 1–40 Hz; 5F/33M; mean age = 12.1	ADHD; HC	4				B↓		(C→D)↑	(43)
Piao et al. (2025)	eyes-closed; 0.5–45 Hz; 5F/29M; mean age = 9.1	ADHD-SP; ADHD-NSP; HC	4		D↓	A↓**			(C→D)↓ (D→C)↓**	(50)
Zarka et al. (2024)	eyes-closed; 1–40 Hz; 10F/10M; mean age: 41.68;	MBSR; WL	4		C↓	C↓	C↓			(51)
Current study	delta	MBCT ADHD; WL ADHD	4						(A→C)↓	
	theta		4	A↑		B↓	A↑		(A→B)↑ (B→D)↓	
	alpha		4	D↑					(C→D)↑	
	Full spectrum		4	B↓			B↓		(B→D)↓	

Some details of each study were mentioned, such as eyes open/closed during EEG recording, the frequency band of analyzed data, gender, and age of participants. The gender and age of the ADHD group of each study are presented in this table. *only observed in the ADHD remission (ADHD-R) group **only in the ADHD without sleep problems (ADHD-NSP) group.

No published study has examined microstates of EEG recordings in ADHD patients following MBCT exposure. However, we might be able to discuss the results of studies that compared EEG microstates of ADHD and healthy controls, or the effects of mindfulness-based interventions on healthy subjects. There are a few studies that fit into these categories. One might expect that if a variable (eg, microstate dynamics) increases in ADHD patients compared to healthy subjects, a treatment should reverse those effects by decreasing the values of that variable. However, no such pattern was observed in our results compared to others. There is an inconsistency with the results of previous studies that compared ADHD with healthy subjects, which is summarized in **Table 3**. Based on this summary, the most obvious change in microstate dynamics between ADHD and healthy controls is the transition probabilities between microstate classes C and D. There are a few variations between previous studies and also with the current study that might explain the inconsistency between these results. The studies summarized in **Table 3** differ in a few aspects, such as the status of their eye (open or closed), the frequency bands, and the age and gender of participants. As the current study shows, there can be a large difference between microstate dynamics in different frequency bands, and this, together with other variations, might explain some of these inconsistent results.

After conducting the microstate analysis on resting-state EEG recordings, we implemented additional analysis on our data to reveal more useful information. At first, we examined the cross-correlation between microstate temporal dynamics and clinical measures. The results indicated a strong correlation within seven pairs of data. Four pairs had positive correlation (**Figures 6A-D**) and three of them had a negative correlation (**Figures 6E-G**). Two strong positive correlations were observed between BRIEF shifting scores and mean GFP in the delta frequency band, one in microstate A and another in microstate D (**Figures 6B, C**). A strong negative correlation between individual explained variance of microstate A in the alpha frequency band was also observed with two clinical measures, the BRIEF behavioral regulation index and global executive composite (**Figures 6E, F**).

Microstates A and B seem to play an important role in the neurophysiological aspects of ADHD patients. The Coverage of microstate B and individual explained variance of microstate A each appeared in two correlations (**Figures 6D-G**). We also observed a significant group×time effect in the temporal dynamics of microstates A and B between groups of study. The individual explained variance and coverage of microstate A increased after MBCT; and the Individual explained variance, duration, and coverage of microstate B decreased after MBCT in our study. The results of other studies also suggest the importance of microstates A and B temporal dynamics in ADHD patients. Férat et al. reported a decrease in duration and coverage of microstate A (17), and Leon et al. reported a decrease in coverage of microstate B (43) in ADHD patients compared to healthy subjects.

The significance of microstates A and B in ADHD patients can also be observed by investigating the relationship between these microstates and brain neural networks. The involvement of the visual cortex in both microstate A and B was detected by previous studies. The association of microstate B with visual regions (among other areas) was reported similarly in a few other studies (52–54). Milz et al. compared the temporal dynamics of EEG microstates during spatial visualization, object visualization, verbalization, and no-task conditions. Their study indicated that microstate A had an increased occurrence, duration, and explained variance during the spatial and object visualization tasks compared to no task and verbalization conditions (55), which can relate microstate A to visual processing. Antonova et al. observed similar results in which the temporal presence of microstate A increased during both visualization and verbalization tasks (56). Finally, the results of an MRI study indicated that the volume of gray matter reduced significantly in the early visual cortex in ADHD patients (57). This shows the importance of the visual cortex as a potential area of dysfunction in ADHD patients.

Some studies have focused on the activation of the temporal cortex and auditory network regarding the underlying neuronal sources of microstate A (52–54, 58). Their similar results indicated that the activity of the auditory network and phonological processing are associated with microstate A. The auditory cortex is connected to a variety of brain structures, such as attentional networks that communicate with auditory processing networks by providing information and receiving precise feedback. This evidence supports the interconnection between attentional and auditory functions (59, 60). There is also evidence of overlap between ADHD and Auditory Processing Disorder (61). In a recent study, Blomberg et al. showed that aberrant interactions between the auditory systems, default mode network, and ventral attention/salience network are linked to inattentiveness in ADHD (62). Serrallach et al. found that adult ADHD patients have a different structural and functional auditory cortex compared to controls (63).

The relationship between the level of participants’ arousal and microstate A has been shown to be a consistent finding. A negative correlation between the subjective scores of sleepiness and coverage and occurrence of microstate A was reported by Ke et al. (64). These results were supported by another study that demonstrated a positive correlation between subjective examination of alertness and the duration of microstate A (56). Several models have been proposed about the origin of ADHD. The hypoarousal theory by Satterfield and Dawson states that an underaroused nervous system is the main cause of ADHD symptoms of hyperactivity, impulsivity, and inattention (65). Reports of other studies also show that low levels of arousal correlate with more severe symptoms of ADHD in adults and children (66–68).

Several studies have investigated the potential associations between cognitive domains and microstate B. Studies with resting-state EEG have reported that microstate dynamics can predict the cognitive performance of individuals. The occurrence of microstate B, for example, is associated with crystallized intelligence (69), potentially reflecting the recruitment of cortical networks involved in semantic memory retrieval, visual conceptual processing, and the consolidation of long-term, acquired knowledge. Du et al. observed a positive correlation between cognitive flexibility inventory scores and the duration of microstate B (70). It is well documented that adults with ADHD, compared to healthy subjects, may show cognitive deficits identified by dysfunctions across all attention modalities, verbal memory, processing speed, reading skills, executive function, arithmetic abilities, and social cognition (71).

Another finding in our study related to the significance of microstates A and B in ADHD patients is the cross-correlation of clinical measures with microstate dynamics. The results of our study, as depicted in **Figure 6**, indicate that almost all correlations happened in microstates A and B. Mean GPF and individual explained variance of microstate A and mean GPF and coverage of microstate B had a strong correlation with clinical measures in our study.

We used machine learning methods, including logistic regression/LR, support vector machine/SVM, and random forest/RF models to predict the treatment response of the MBCT program using microstate dynamics. Our purpose was to see if the models could distinguish high-benefitters from low-benefitters, which were initially categorized by increment change in clinical measures (CAARS and OQ-42.5) across the randomized trial period. Notably, the logistic regression/LR model was able to perform better than other models in reaching a high accuracy (83%), which was also higher than other studies that used machine learning techniques to predict the outcome of mindfulness-based interventions. Myers et al. have implemented a random forest/RF model to predict the response of MBCT for suicide prevention and reached an accuracy of 0.7 utilizing clinical and neurocognitive task data (72). In another study, Dethoor et al. used the support vector machine/SVM model to predict treatment response of MBCT in patients with depressive symptoms. They constructed a support vector machine/SVM model with two clinical measures (Beck Depressive Inventory and Five-Facet Mindfulness Questionnaire), which had a sensitivity of 0.79 and a specificity of 0.71 (73). Their results are comparable with our results of using the support vector machine/SVM method with 0.66 sensitivity and 0.77 specificity.

Our study includes limitations to be addressed. First, our sample could benefit with a larger number for machine learning applications, that generally require a high sample size to ensure high generalizability. However, our sample size is comparable with other studies such as Férat et al. (61 healthy, 61 ADHD in dataset 1 and 22 healthy and 22 ADHD in dataset 2) (17), Luo et al. (54 healthy, 54 ADHD-I, and 53 ADHD-C) (44), and Wu et al. (28 healthy, 50 ADHD) (16). Second, we compared the effects of MBCT with a passive wait-list, opposed to active, control group that did not participate in any focused activity. This is not inherently problematic for initial mechanistic exploration, since passive controls allow for a clear comparison of intervention versus no intervention when prior mechanistic models/data are limited. Passive control designs are not necessarily weaker, for example, in cognitive training research, a recent meta-analysis (from 1, 524 studies) reported no consistent differences in outcomes between active and passive controls (74), and that passive groups may offer a more reliable baseline for retest effects across studies. Nevertheless, future studies could increase mechanistic specificity by including active control groups, such as cognitive training (e.g. CBT), health enhancement programs, or psychoeducation, to further clarify the specific working pathways involved in MBCT for ADHD. Third, the overlap of ADHD with other conditions that may produce similar neurophysiological patterns needs to be addressed in future studies. For example, a relatively recent study showed that patients diagnosed with ADHD share common neural deficits with spontaneous mind wandering in neurotypical individuals (75). Furthermore, although the value of feature extraction using resting-state EEG data has shown to be important, it would be beneficial to examine these features using neurophysiological markers (e.g. ERP, ERSP, ITC). Previous studies report a relationship between these neural outcomes and brain generators associated with adult ADHD (76, 77). Examination of event-related microstates is currently underway within our group to further elucidate these neurophysiological dynamics, which we anticipate will extend and refine the resting-state findings presented here.

## Conclusion

This study is the first to demonstrate that mindfulness-based cognitive therapy (MBCT) produces measurable changes in resting-state EEG microstate dynamics in adults with ADHD. MBCT led to significant improvements in clinical outcomes and modulated the temporal dynamics of specific microstate classes across multiple frequency bands, suggesting neurophysiological mechanisms underlying therapeutic gains. The results of our research highlight the significance of microstates A and B in individuals with ADHD, which have also been shown to be modulated in other neuropsychiatric disorders and are associated with aberrations in visual and auditory processing. Furthermore, our machine learning model successfully predicted the treatment response of the MBCT program, pointing to the potential of personalized, data-driven approaches in ADHD treatment. Future research may explore microstate dynamics across ADHD subtypes and age groups to deepen mechanistic insights and guide targeted mindfulness-based interventions.

## Data Availability

The raw data supporting the conclusions of this article will be made available by the authors, without undue reservation.
